# Evaluation of Quality of Life and Treatment Satisfaction in Newly Diagnosed Cutaneous T-Cell Lymphoma Patients

**DOI:** 10.3390/cancers16050937

**Published:** 2024-02-26

**Authors:** Rosanne Ottevanger, Sylvia van Beugen, Juliette M. Kersten, Andrea W. M. Evers, Maarten H. Vermeer, Rein Willemze, Koen D. Quint

**Affiliations:** 1 Department of Dermatology, Leiden University Medical Center, 2333 ZA Leiden, The Netherlandsm.h.vermeer@lumc.nl (M.H.V.);; 2 Health, Medical and Neuropsychology Unit, Institute of Psychology, Leiden University, 2311 EZ Leiden, The Netherlands

**Keywords:** cutaneous T-cell lymphoma, quality of life, mycosis fungoides, Sezary syndrome, treatment expectations, treatment satisfaction

## Abstract

**Simple Summary:**

This study provides an insight into newly diagnosed patients with MF on patients’ expectations, quality of life, and treatment satisfaction. These data can be used for adequate expectation management and provide a rationale for further evaluation of treatment regimens in these patients.

**Abstract:**

Background: Little is known about the impact of MF on quality of life (QoL) in newly diagnosed patients. Objectives: To describe the impact of the MF diagnosis on QoL, patient expectations, and treatment satisfaction over the first 6 months after diagnosis. Methods: Outcomes of this prospective cohort study of newly diagnosed MF patients conducted between 2020 and 2022 at the Leiden University Medical Center included the Skindex-29, RAND-12 Health Survey, degree of itch, pain, and fatigue (Visual Analogue Scale (VAS)), patient expectations, and Client Satisfaction Questionnaire-8 (CSQ-8), measured at baseline and after six months. Results: A total of 28 patients with MF were included. At baseline, 66% (n = 18) “strongly-totally” expected positive effects of the treatment. At the time of diagnosis, 28% of the patients (n = 8) were moderately to severely affected. There was no statistical change in the Skindex-29 score sum score (20 [10–34] vs. 20 [9–36]; *p* = 0.81) or in the other three subdomains, the RAND-12 scores, and the VAS itch, pain, and fatigue over time. Treatment satisfaction was high overall. Conclusion: Despite that the newly diagnosed MF patients anticipate a positive treatment effect, few improvements in QoL and symptom reduction were found. These data can be used for adequate expectation management and provide a rationale for further evaluation of treatment regimens in these patients.

## 1. Introduction

Mycosis fungoides (MF) is the most frequent subtype of primary cutaneous T-cell lymphoma (CTCL). CTCL are a heterogeneous group of diverse entities, such as Sezary syndrome and aggressive epidermotropic cutaneous CD8+ lymphoma, that usually present in the skin with no evidence of extracutaneous disease at the time of diagnosis [1,2]. Mycosis fungoides can be subdivided into classical MF (cMF) and folliculotropic mycosis fungoides (FMF), a variant with distinctive clinical and pathological features [3]. There are different disease stages of MF, varying from localized patches and plaques to ulcerative tumors and erythroderma [1,4]. While it is still a rare disease, the current incidence of MF is rising and is currently estimated at 0.46 per 100,000 persons [5].

The prognosis of MF is greatly dependent on the disease stage. Also, the diverse manifestation results in a broad range of symptoms and varies from patient to patient, but symptoms can be experienced as very severe, especially in the case of profound itching [6,7,8]. It is well known that the quality of life (QoL) in patients with MF is significantly lower than in the general population [9,10,11,12]. Previous studies even compare the impact of late-stage CTCL with diseases like end-stage renal disease and diabetes [6,13,14].

In addition, it has been previously reported that patients with a more recent diagnosis of CTCL were more affected in the symptom and functioning domains and had more intense itching [7,15]. On top of this, there is a significant impact of receiving the diagnosis, often after a diagnostic delay of 2–10 years from the onset of symptoms [16].

Only the PROCLIPI study has described health-related QoL in relatively newly diagnosed patients, but in this study, these patients were included within a timeframe of 6 months after the diagnosis, and not immediately after they received the diagnosis [17]. In addition, patients’ expectations have not been explored to date. It could be that the QoL and degree of itching improves significantly in the first months after the diagnosis. Awareness of the course of QoL and patients’ expectations are necessary for clinicians in order to properly counsel patients and provide realistic expectations. 

This study aims to describe the impact of the MF diagnosis on QoL, patients’ expectations, the course of QoL, and treatment satisfaction over the first 6 months. Also, differences between cMF and FMF are explored. 

## 2. Materials and Methods

A prospective cohort study was performed between May 2020 and January 2022 of all newly diagnosed cMF and FMF patients aged >18 years old referred to the dermatology department of the Leiden University Medical Center (LUMC) that were willing and able to provide a formal written consent. The LUMC is the national referral center in the Netherlands for CTCL patients. This study was approved by the institutional medical ethical review board (N20.052). Patients were included in case they received the diagnosis MF and filled out baseline questionnaires within one month after the diagnosis. All included patients were diagnosed according to the clinicopathologic criteria of the World Health Organization-European Organization for Research and Treatment of Cancer [3]. In case of MF, disease stages IA-IIA were defined as early stage and ≥IIB was considered as late stage. FMF was classified as early stage or late stage. Disease progression was defined as progression from early- to late-stage disease. 

Patients were asked to complete baseline questionnaires related to dermatology specific QoL, generic health-related QoL, and treatment expectations as we previously described [18]. After six months, these questionnaires were repeated, except for treatment expectations, and treatment satisfaction was explored. 

### 2.1. Outcome Measurements

The primary outcome was the potential change in dermatology specific QoL over time. Secondary outcomes were generic health-related QoL, treatment expectations, and treatment satisfaction. In addition, baseline characteristics, such as age, sex, comorbidities using the Charlson Comorbidity Score (CCI), and if available, disease progression within the study period, were extracted from the patients’ medical records. 

### 2.2. Skindex-29

The Skindex-29 is a very frequently used questionnaire that specifically addresses the QoL in patients with skin disease [19]. The 29-item questionnaire is also the most frequently used QoL instrument in QoL studies in CTCL patients [6]. Individual items measure the frequency of affected QoL on 5 levels using a 5-point Likert scale ranging from 1 (‘never’) to 5 (‘all the time’). The individual items can be categorized in a symptoms domain, emotions domain, functional limitations domain, and as a total score. Each of the domains have different cut-off points, as proposed by Prinsen et al. These are ≥39, ≥42, and ≥52 for mild, moderately, and severely impaired for the symptoms domain; ≥24, ≥35, and ≥39 for the emotions domain; and ≥21, ≥32 and ≥37 for the functional limitation domain, respectively. The cut-off points for the total Skindex-29 score is ≥25 for mild, ≥32 for moderate, and ≥44 for severe impairment. Scores below the lowest cut-off points for mildly impaired were regarded as unaffected [20].

### 2.3. RAND-12

The RAND-12 Health Survey (RAND-12) is derived from the RAND-36 questionnaire. RAND-12 is used to measure general health-related QoL and results in a physical component score (PCS) and mental component score (MCS). The two domain scores range from 0 to 100, with higher scores indicating a better health status. The minimally, clinically important difference is considered in case of a 3-to-5-point difference over time [21].

### 2.4. Itch, Pain, and Fatigue

The degree of itch, pain, and fatigue in the last 4 weeks was reported with a visual analogue score (VAS) (0–10) [22].

### 2.5. Expectations Questionnaire

To assess treatment expectations, a 6-item unvalidated questionnaire, also used in a previous study by Ottevanger et al., was used [18]. This questionnaire explores expectations related to the expected effect of the treatment on pain, time consumption, mood, energy, the anticipated treatment effect, and anticipation of adverse events due to the treatment. The results of this questionnaire, because there are not yet validated, are used for descriptive analyses only. 

### 2.6. Client Satisfaction Questionnaire (CSQ)

The CSQ was used to establish treatment satisfaction after six months [23]. The 8-item questionnaire results in eight scores that range from 25 to 100. No clinically correlated categories can be derived from the total score; items are analyzed separately. 

### 2.7. Statistical Analysis

Statistical analysis was performed with IBM SPSS statistics 25, Armonk, NY, and reported following the Strengthening the Reporting of Observational studies in Epidemiology (STROBE) guidelines [24]. All analyses were performed according to corresponding instrument manuals. The normality of data distribution was tested with the Kolmogorov–Smirnov test. Continuous data were reported as median and interquartile range (IQR) due to the non-normal distribution and categorical data as number and percentage (%), as applicable. To establish potential improvement or worsening over time, the Wilcoxon rank test was performed.

Statistical tests were performed in IBM SPSS statistics version 29.0 for the total group of patients and to test for differences between cMF and FMF. The correlations with degree of itch and fatigue and Skindex-29 scores were assessed using the Pearson R correlation analysis. A coefficient of ≤0.19 was considered very weak, 0.20–0.39 weak, 0.40–0.59 moderate, 0.60–0.80 strong, and 0.80–1 very strong. 

## 3. Results

### 3.1. Study Population

In total, 28 patients with MF were included with a median age of 65 years [50–72] (Table 1). Most patients were diagnosed with cMF (n = 21 (75%)) and an early-stage disease (n = 26 (93%)). Two patients (7%) experienced disease progression from early- to late-stage disease during the study period (one cMF and one FMF case). The median overall CCI score was 1 [0–3]. There were no statistical differences in baseline characteristics between cMF patients and FMF patients (Table 1). All patients survived the follow-up period and there was no loss to follow-up.

### 3.2. Patient Expectations

Most patients (82% (n = 22) expected to not experience any pain (Table 2). Approximately one-third “strongly-totally” expected the treatment of their disease to be time consuming. All patients “Not at all-somewhat” expected a decrease in their energy levels. Many expected the treatment to have positive effects on their symptoms, with 33% (n = 9) expecting some effects of their treatment on symptoms, 44% (n = 12) strongly expecting treatment effects, and 22% (n = 6) totally expecting treatment effects. Only one patient (4%) strongly expected adverse events. The remaining patients (96%) “not at all” to “somewhat” expected side effects.

### 3.3. Skindex-29

During the study period, most patients (57% (n = 16) at baseline and 61% (n = 17) after six months) were unaffected on the total Skindex-29 score (Table 3). There was no statistical change from baseline during the study period in the total Skindex-29 score sum score (20 [10–34] vs. 20 [9–36] with *p* = 0.81) or in the other three subdomains. At baseline, 28% (n = 8) were moderately to severely affected. After six months, this was relatively unchanged, namely, 32% (n = 9). Regarding the symptoms domain categories, 21 patients (75%) remained stable, 4 (14%) improved, and 3 (11%) worsened. This was almost identical in the emotions domain. In the functioning domain, 86% (n = 24) remained stable and 14% (n = 4) improved. For the total Skindex-29 categories, 79% (n = 22) remained stable, 14% (n = 4) improved, and 7% (n = 2) worsened. The two patients that experienced disease progression to a late-stage disease did not worsen regarding their total Skindex-29 or symptoms score. 

FMF patients had a higher total Skindex-29 score (42 [10–50] vs. 16 [9–25]; *p* = 0.03) and higher function domain score (41 [10–50] vs. 4 (0–9); *p* = 0.02) than cMF patients at baseline. No baseline differences between FMF and cMF patients were found for the symptoms and emotions domain. At follow-up, FMF patients scored statistically higher on all Skindex-29 domains compared to cMF patients (53 [36–71] vs. 21 [14–46], *p* = 0.04 for symptoms; 40 [18–58] vs. 20 [10–29], *p* = 0.03 for emotions; 35 [8–62] vs. 6 [0–13], *p* < 0.01 for functioning; 37 [22–59] vs. 14 [8–23], *p* = 0.01 for the total skindex-29 score). Within the cMF group, there was no statistically significant change over time for the total Skindex-29 score or any of the other domains. The same was also found in the FMF group. 

### 3.4. RAND-12

The RAND-12 PCS after six months did not statistically change from baseline (55 [46–57] to 54 [37–55], *p* = 0.13) (Table 3). This was also the case for the MCS (50 [37–55] to 51 [39–54], *p* = 0.68). Within this timeframe, 54% (n = 15) remained clinically stable according to the minimally, clinically important difference regarding their PCS score; 18% (n = 5) improved; and 29% (n = 8) worsened. For the MCS, 50% remained stable, 29% (n = 8) improved, and 21% (n = 6) worsened. There were no differences at baseline or follow-up for the median PCS or MCS between cMF and FMF (*p* = 0.16, *p* = 0.17, *p* = 0.09, and *p* = 0.14, respectively). 

### 3.5. Itch, Pain, and Fatigue

The median itch, pain, and fatigue VAS scores at baseline were 2.5 [1.0–5.8], 1 [0–2.0], and 4 [1.3–6.8] (Table 3). These also did not statistically change over time (*p* = 0.75, 0.92, and 0.74, respectively). There was a statistically significant difference at baseline between cMF and FMF patients, with FMF patients experiencing more itching (VAS 2 [1–4] vs. 6 [2–10]; *p* = 0.042) and fatigue (VAS 3 [1–5.5] vs. 7 [5–8]; *p* < 0.01). This statistically significant difference remained after 6 months with a median VAS itch score of 1 [0–5] in the cMF group and median score of 8 [1–9] in the FMF group (*p* = 0.017). This was also the case for fatigue (median VAS fatigue 3 [1–4.5] vs. 8 [4–9], *p* = 0.017). There were no statistical differences between baseline and follow-up for itch or fatigue within the cMF or FMF group.

The VAS itch at baseline was a strong predictor for the baseline Skindex-29 symptoms (R 0.70; *p* < 0.01) and total skindex-29 score (R 0.60; *p* < 0.01), while only moderate for the emotions (R 0.49; *p* < 0.01) and functioning domains (R 0.44, *p* = 0.02). The VAS fatigue was only moderately predictive for all Skindex-29 domains. 

### 3.6. Treatment Satisfaction

The overall treatment satisfaction was high, with 39% (n = 11) being “mostly satisfied” and 50% (n = 14) being “very satisfied” (Table 4). Only 11% (n = 3) were “indifferent or mildly dissatisfied”. Almost all patients were satisfied with the quality (90% “good-excellent”), the type of treatment they received (89% “generally-definitely”), and the support they received with the treatment to deal with their disease (89% “somewhat-helped a great deal”).

## 4. Discussion

The results of this study show that although most patients are unaffected regarding their Skindex-29 scores, a significant proportion experience a reduced QoL without a significant improvement over time. Although patients have high expectations that treatment would result in symptom reduction, this reduction was not found in the Skindex-29 scores. This group could require extra counseling. Meanwhile, treatment satisfaction was high despite that no improvement in QoL or reduction in itch and fatigue was seen, especially in FMF patients. 

This is one of the first prospective studies on the clinical course regarding QoL in the first 6 months after receiving diagnosis while also exploring patients’ expectations and evaluating treatment satisfaction. Only the PROCLIPI study (2021) has previously described QoL in newly diagnosed patients. The median Skindex-29 scores in our study are similar to those found in the PROCLIPI study by Quaglino et al. [17]. However, a follow-up measurement was available in less than half of their patients (56 out of 121). In addition, patients were considered newly diagnosed if they had received a diagnosis less than 6 months prior to filling out questionnaires. In contrast to our study, the study of Quaglino et al. did find a small but statistically significant reduction in the total sum score of 24 to 19 after a median follow-up period of 13 months (range 1–43) [17]. Although this was a statistically significant decrease, it remains questionable whether this is of any clinical importance. 

As mentioned before, it has been described that patients with a more recent diagnosis (<2 years vs. ≥2 years) had a worse QoL [7]. It could also be hypothesized that it takes longer than the follow-up period of 6 months in this study for first- or second-line therapies to have an effect on symptom reduction and improve QoL. This hypothesis is also confirmed by Quaglino et al., who showed that 75% of patients have persistent skin lesions after their first-line treatment [17]. Complete responses of topical therapies are often not or only temporarily seen in patients with early-stage disease [25]. This also reiterates the chronic aspect of the disease, even in newly diagnosed patients, especially when patients have the expectancy of a strong–total effect of the treatment on their symptoms, while one third of the patients remains to experience a significantly reduced QoL over time. These expectations have to be addressed appropriately in order to prevent unrealistic expectations and increase adequate coping mechanisms [26]. Although this seems to be adequately addressed with high levels of treatment satisfaction being reported in this cohort, addressing the different domains of QoL during the treatment course is very important to provide personalized, if needed multidisciplinary, care for every patient [27]. In other chronic diseases, this has shown to yield some positive results regarding QoL [28].

Despite that the majority of patients had an early-stage disease, just over one-third of the patients experienced a moderate–severe impaired QoL on baseline. These results are in line with previous studies on early-stage MF patients, with others demonstrating that a significant proportion of patients reporting moderate–severe impairment on QoL [15]. This study also found itching, fatigue and impact on overall QoL to be significantly worse in FMF patients compared to cMF patients. Also, a reduction in itching and fatigue was not seen within the first 6 months. This could be due to the known poor responsiveness to skin-directed therapies [17,29,30]. 

This study had some limitations. There was a small sample size, and treatment regimens were not taken into account. The small sample size resulted in limited generalizability regarding the cMF vs. FMF comparisons. The different therapies that patients received during the study period were not taken into account. However, future studies should also focus on relating the effect of treatment on the QoL for the different CTCL subtypes and stages. 

## 5. Conclusions

Overall, this study showed that a significant proportion of patients experience an impaired QoL and that few improvements in QoL are seen within the first six months in newly diagnosed patients with MF while having high expectations of symptom reductions. These data can be used for adequate expectation management and provide a further rationale for a more in-depth analysis of treatment regimens in newly diagnosed MF patients and their effect on QoL and symptom reductions. In addition, the lack of improvement could require extra counseling in patients.

## Figures and Tables

**Table 1:** Patient characteristics.

Patient Characteristics		Total n = 28	MF n = 21	FMF n = 7
Age		65 [50–72]	66 [54–75]	54 [35–66]
Gender	Male	16 (57%)	13 (62%)	3 (43%)
CCI		1 [0–3]	0 [0–2]	2 [0–3]
	0	13	11	2
	1	5	5	0
	2	3	1	2
	3	6	4	2
	4	0	0	0
	5	1	0	1
Disease stage	Early stage	26 (93%)	21 (100%)	5 (71%)
	Late stage	2 (7%)	0	2 (29%)

Numbers are displayed as number (%) or median [interquartile range]. CCI, Charlson Comorbidity Index; MF, Mycosis fungoides; FMF, Folliculotropic Mycosis fungoides.

**Table 2:** Patient expectation at baseline on six different domains.

Domain	Expected Level	n (%) (n = 27)
Pain	Not at all	22 (82%)
	Somewhat	5 (19%)
	Strongly	-
	Totally	-
Time consumption	Not at all	8 (30%)
	Somewhat	11 (41%)
	Strongly	6 (22%)
	Totally	2 (7%)
Mood	Not at all	23 (85%)
	Somewhat	4 (15%)
	Strongly	-
	Totally	-
Energy	Not at all	21 (78%)
	Somewhat	5 (19%)
	Strongly	1 (4%)
	Totally	-
Treatment effect on symptoms	Not at all	-
	Somewhat	9 (33%)
	Strongly	12 (44%)
	Totally	6 (22%)
Adverse events	Not at all	14 (52%)
	Somewhat	12 (44%)
	Strongly	1 (4%)
	Totally	-

Numbers are displayed as number (%).

**Table 3:** Skindex-29, RAND-12, and itch, pain, and fatigue VAS scores at the start and end of study period, including median scores.

Outcomes		Baseline (n = 28)	FU (n = 28)	*p*
**Skindex-29 categorical scores**				
Symptoms	Unaffected	16 (57%)	17 (61%)	
	Mildly	2 (7%)	-	
	Moderately	5 (18%)	4 (14%)	
	Severely	5 (18%)	7 (25%)	
Emotions	Unaffected	14 (50%)	15 (54%)	
	Mildly	7 (25%)	5 (18%)	
	Moderately	1 (4%)	2 (7%)	
	Severely	6 (21%)	6 (21%)	
Functioning	Unaffected	20 (71%)	21 (75%)	
	Mildly	2 (7%)	3 (11%)	
	Moderately	1 (4%)	1 (4%)	
	Severely	5 (18%)	3 (10%)	
Total	Unaffected	18 (64%)	19 (68%)	
	Mildly	2 (7%)	-	
	Moderately	4 (14%)	6 (21%)	
	Severely	4 (14%)	3 (11%)	
Skindex-29 total scores *				
	Symptoms (0–100)	32 [17–46]	30 [15–53]	0.75
	Emotions (0–100)	24 [15–36]	21 [10–21]	0.43
	Functioning (0–100)	6 [1–31]	6 [6–24]	0.66
	Total (0–100)	20 [10–34]	20 [9–36]	0.81
**RAND-12**				
PCS (0–100)		55 [46–57]	54 [37–55]	0.13
MCS (0–100)		50 [37–55]	51 [39–54]	0.68
**VAS**				
Itch (0–10)		2.5 [1.0–5.8]	1 [1.0–7.8]	0.75
Pain (0–10)		1 [0–2.0]	1 [0–2.0]	0.92
Fatigue (0–10)		4 [1.3–6.8]	3 [1.0–5.0]	0.74

Numbers are displayed as number (%) or median [interquartile range]. PSC, Physical Component Score; MCS, Mental Component Score; VAS, Visual Analogue Scale; M1, baseline measurement; FU, measurement after 6-month follow-up. * Mean differences with corresponding IQR per Skindex-29 domain.

**Table 4:** Treatment satisfaction as measured with the CSQ-8.

Domain	Evaluation	n (%)
Quality of treatment	Poor	-
	Fair	3 (11%)
	Good	17 (61%)
	Excellent	8 (29%)
Type of treatment	No, definitely not	1 (4%)
	No, not really	2 (7%)
	Yes, generally	16 (57%)
	Yes, definitely	9 (32%)
Met needs	None of my needs have been met	2 (7%)
	Only a few of my needs have been met	1 (4%)
	Most of my needs have been met	14 (50%)
	Almost all of my needs have been met	11 (39%)
Recommend to a friend	No, definitely not	-
	No, not really	2 (7%)
	Yes generally	11 (39%)
	Yes, definitely	15 (54%)
Amount of help	Quite dissatisfied	-
	Indifferent or mildly dissatisfied	-
	Mostly satisfied	22 (78%)
	Very satisfied	6 (22%)
Deal with problems	No, it seemed to make things worse	-
	No, not really	3 (11%)
	Yes, somewhat	12 (43%)
	Yes, helped a great deal	13 (46%)
Overall satisfaction	Quite dissatisfied	-
	Indifferent or mildly dissatisfied	3 (11%)
	Mostly satisfied	11 (39%)
	Very satisfied	14 (50%)
Come back	No, definitely not	-
	No, I don’t think so	2 (7%)
	Yes, I think so	7 (25%)
	Yes, definitely	19 (68%)
Total CSQ-8 score (25–100)		85 [76–96]

Numbers are displayed as number (%) or median [interquartile range].

## Data Availability

Data will be available upon reasonable request via the corresponding author.
